# Supplementary material for: Specific features and assembly of the plant mitochondrial complex I revealed by cryo-EM
Source: Nat Commun. 2020 Oct 15;11:5195. doi: 10.1038/s41467-020-18814-w (PMC7567890; doi:10.1038/s41467-020-18814-w)
Supplement: Supplementary file 1 — Supplementary Information [file 41467_2020_18814_MOESM1_ESM.pdf]

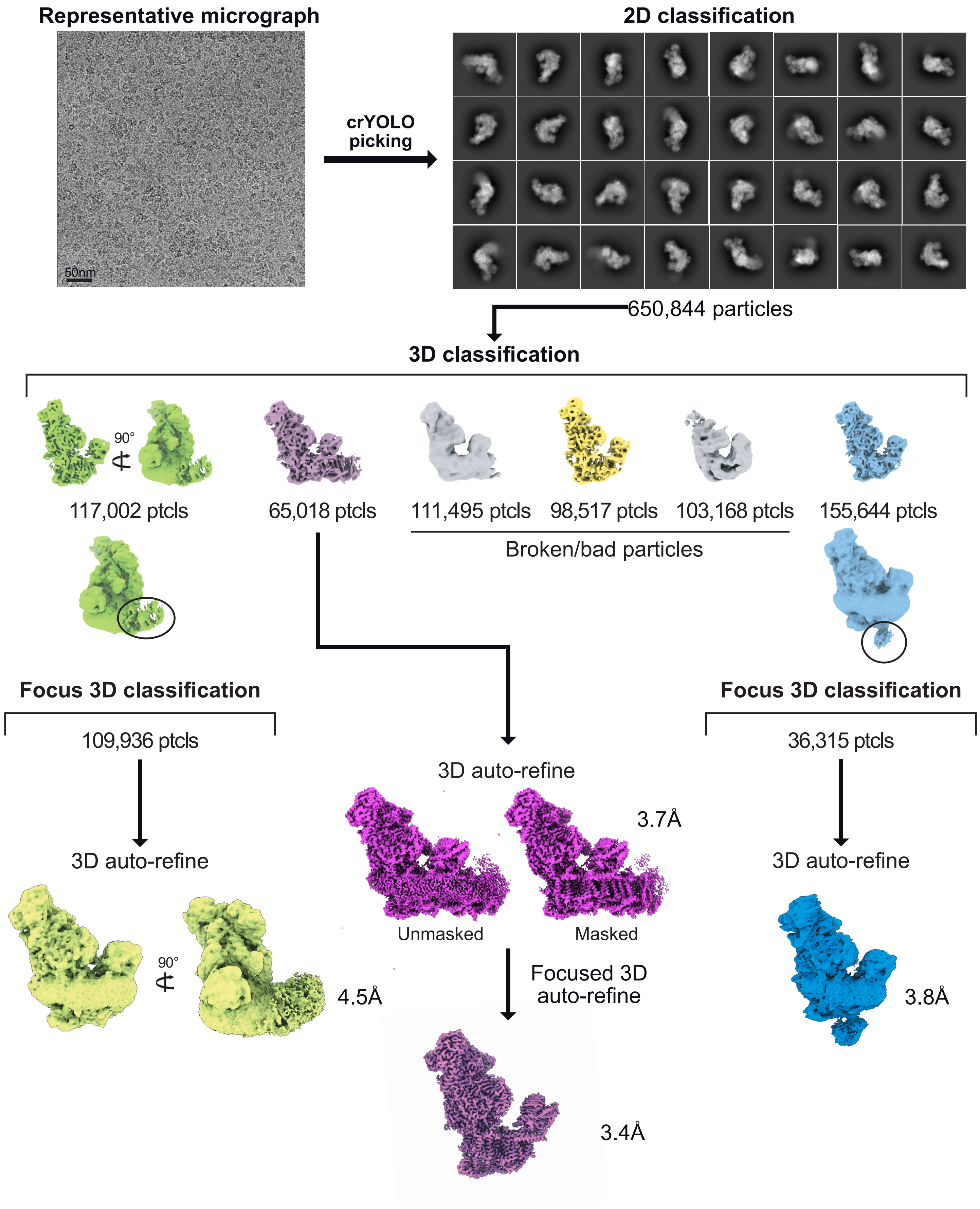

Supplementary Figure 1. Data processing workflow

Graphical summary of the processing workflow described in Methods, with 2D classes presented in **a** and 3D processing and refinement presented in **b**. Density in purple represent the mature and focused reconstructions, density in blue represent the assembly intermediate. During 3D classification, two other type of classes were observed. The first one is presented here as the green density maps, corresponding to a complex where the whole P_D module is shifted. The second are classes corresponding to complexes where the whole P_D module is missing but where no additional density (GLDH) could be observed, listed here as broken/bad particles. For the complexes where the whole P_D module is shifted, the resolution of the P_D module did not allow to clearly understand what the complex was undergoing. Our hypothesis is that these complexes correspond to complex I falling apart, and would represent an intermediate state with the broken complexes.

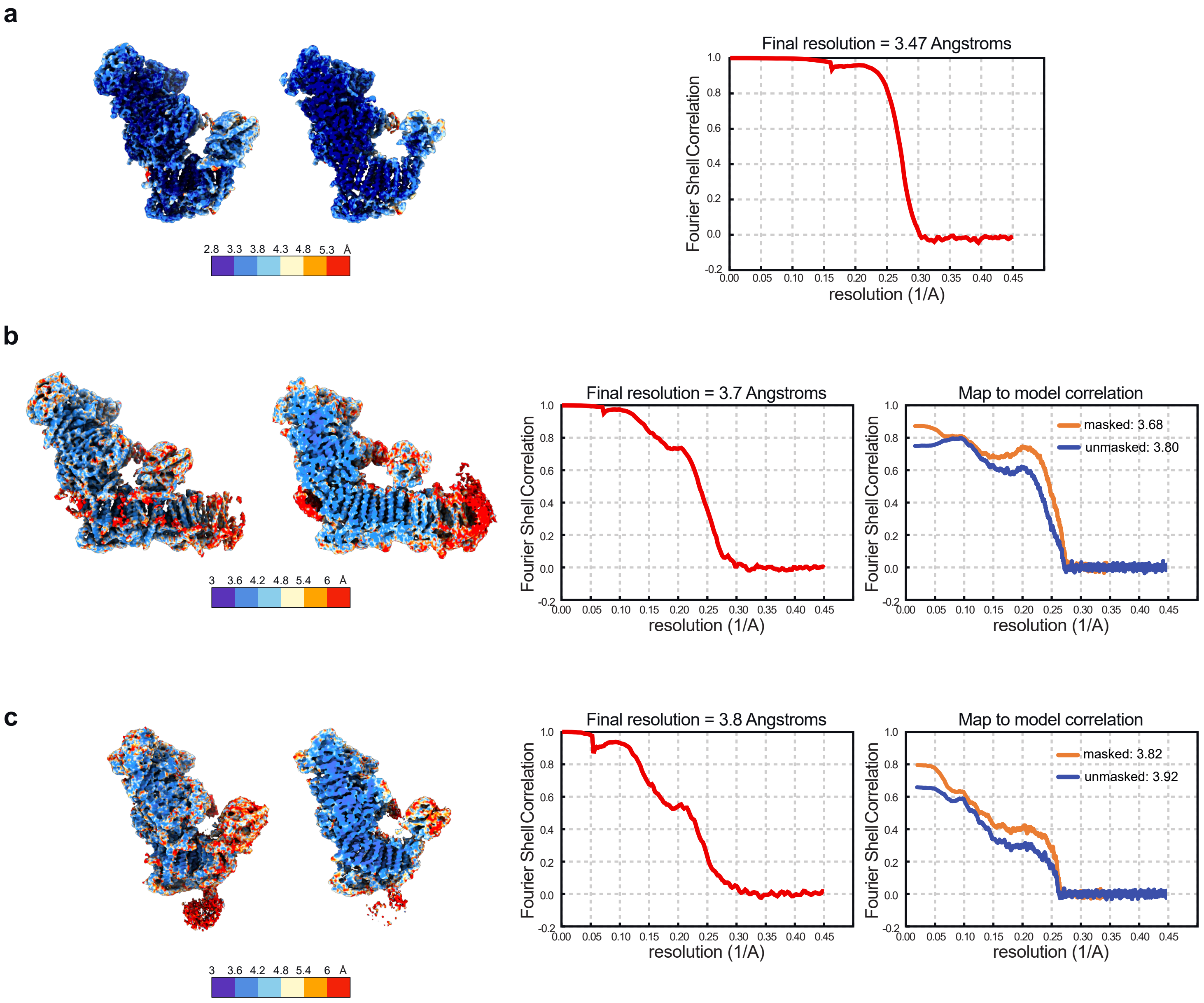

Supplementary Figure 2. Local resolution of the final reconstructions

Local resolutions of both the full **a** and focused **b** complex I as well as the GLDH assembly intermediate complex **c** are shown. The maps are colored by resolution, generated using ResMap[34]. Maps are also shown in cut view. For both reconstructions FSC plots (output from RELION[14]) are displayed for resolution estimation. Map to model FSC are also shown (out from PHENIX[35] validation). CC value of the model versus map for the full complex is 0.71 and 0.47 for the assembly intermediate. The maps resolution were calculated at the 0.143 threshold.

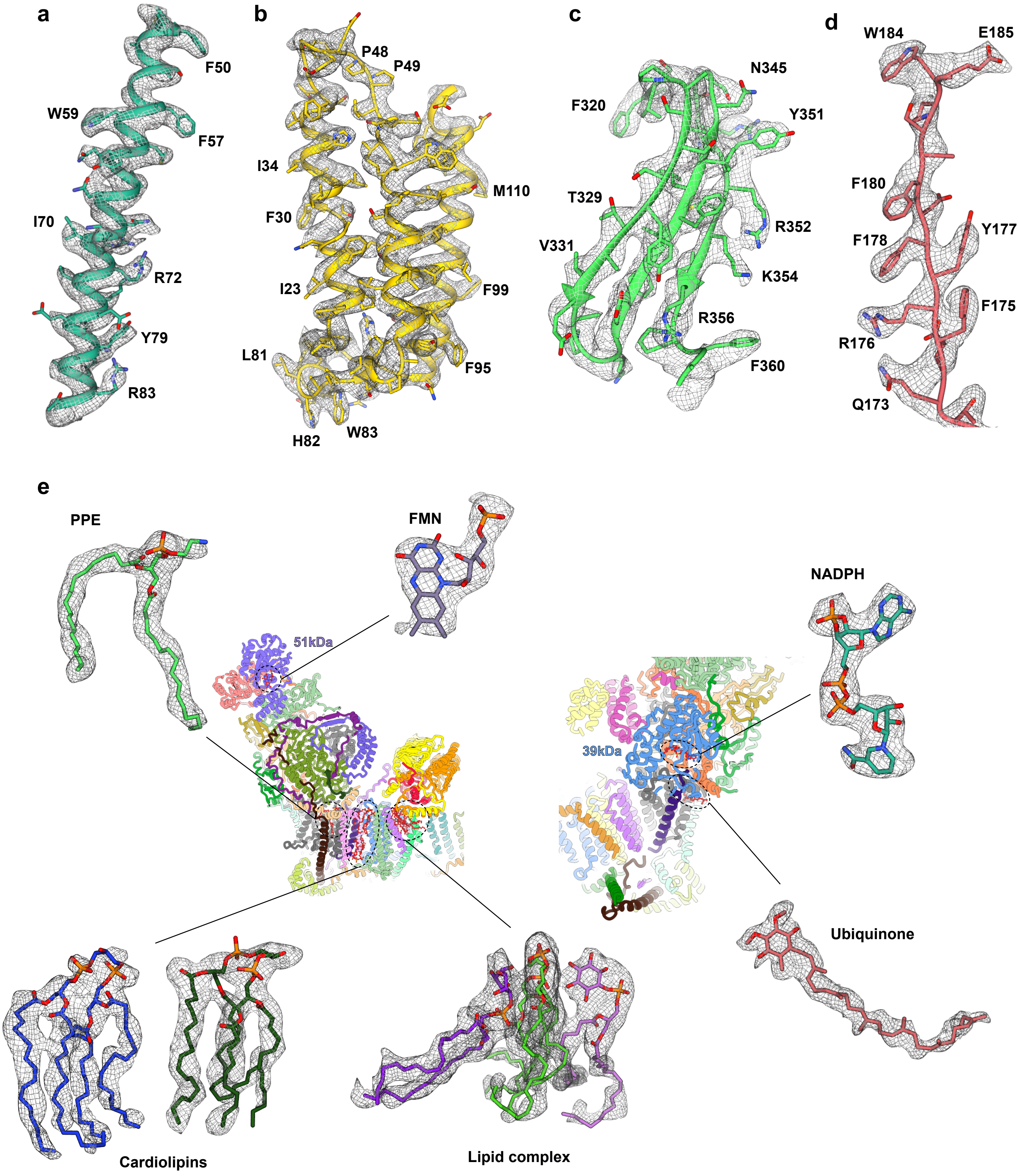

Supplementary Figure 3. Representative cryo-EM densities

a-d Protein densities with **a** showing a helical segment of protein B16.6, **b** several helical segments of protein Nad2, **c** beta strand segments of protein Nad7 and **d** segment of protein Nad1 without particular secondary structure. **e** Lipids and additional ligands identified are presented in their respective densities with their position indicated on the atomic model of complex **I**. The carbonic anhydrase lipid complex is composed of a cardiolipin, in green, and two phosphatidylinositols in purple and orchid. PPE stands for phosphatidylethanolamine.

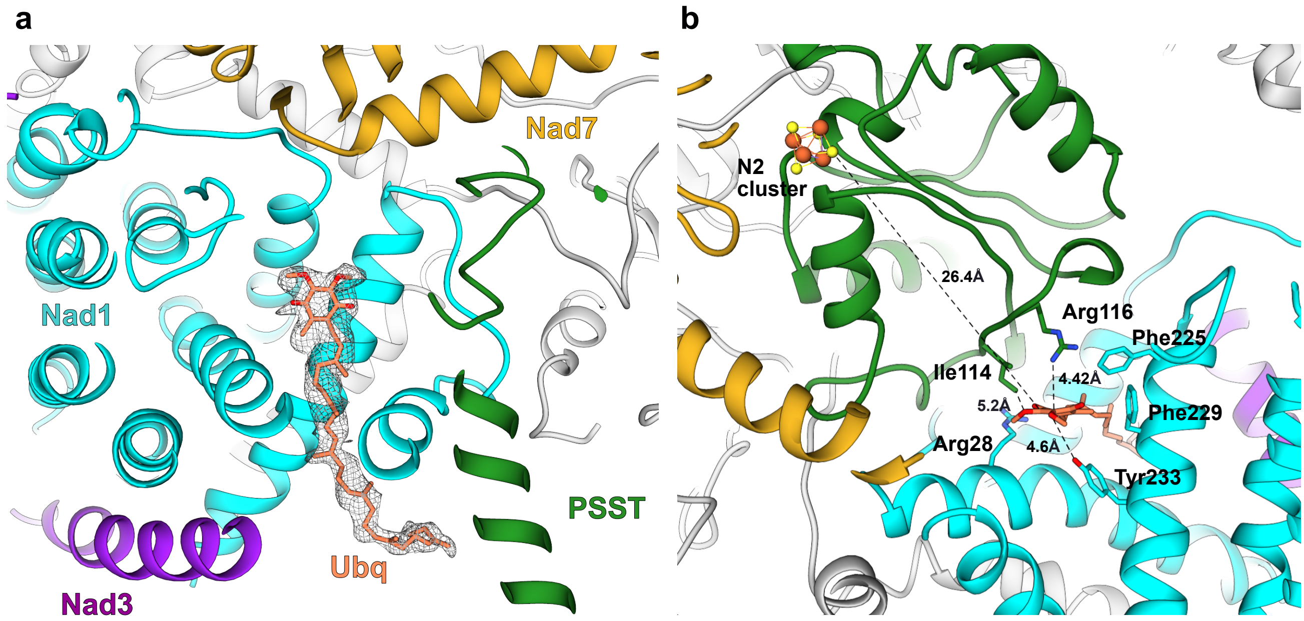

Supplementary Figure 4. Ubiquinone binding site

a Overall view of the ubiquinone pocket mainly formed by Nad1 and PSST. The ubiquinone is shown in its density. **b** Distances between the ubiquinone head group and surrounding residues as well as to the N2 iron-sulfur cluster is shown. The position and coordination of ubiquinone are similar to what has been observed in the *Y. lipolytica* mitochondrial complex I (e.g here 26.4 Å between N2 and the ubiquinone compared to 26.7 Å in yeast)[8].

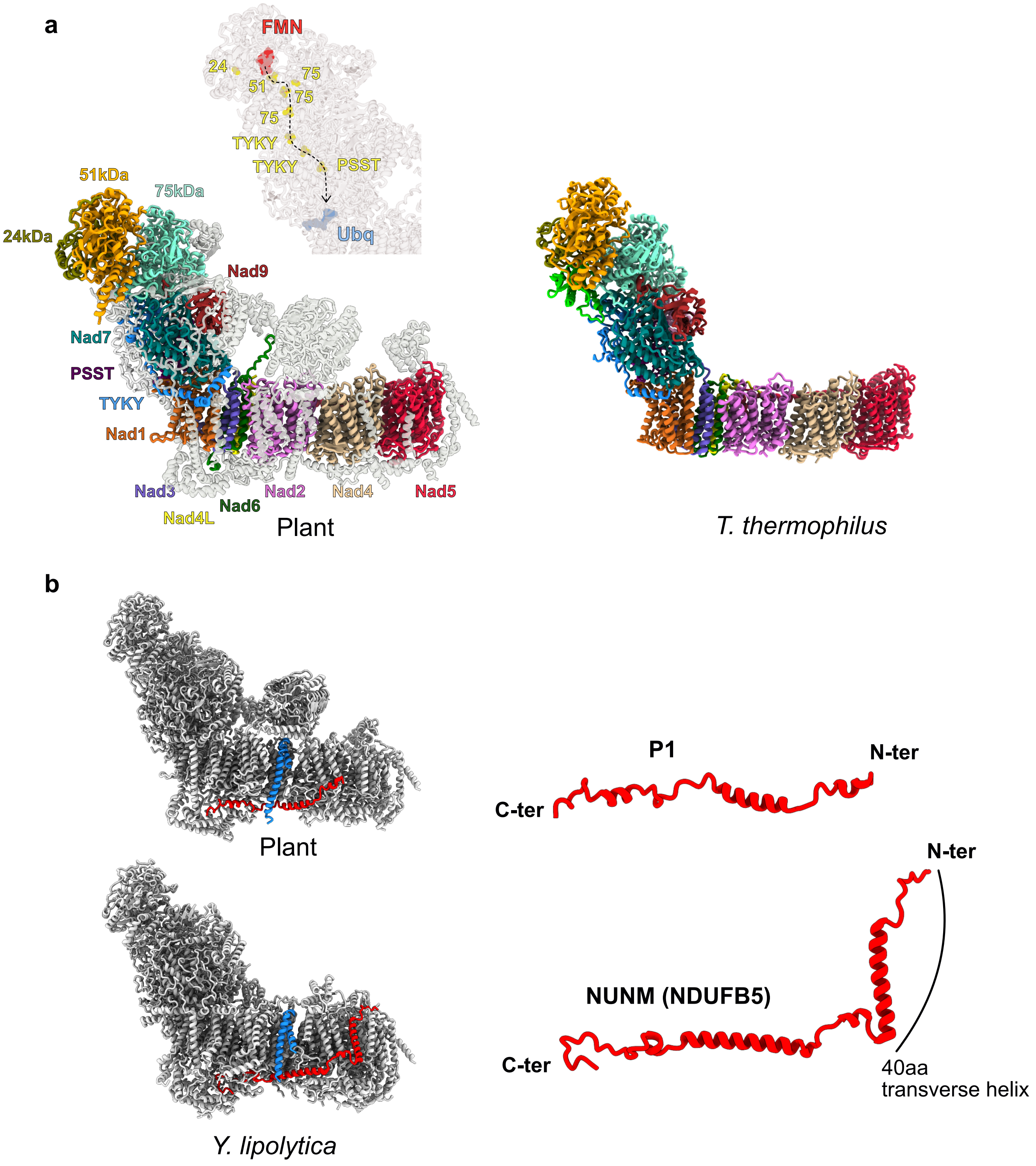

<u>Supplementary Figure 5. Plant mitochondrial complex I comparison with bacterial and other mitochondrial complex I</u>

a Plant mitochondrial complex I is shown compared to its bacterial homologue (*T. thermophilus* model PDB:4HEA). The 14 core subunits are colored in different shades and their names are indicated. Mitochondria and plant specific proteins are shown in gray and transparent. Electron path in matrix arm from FMN to ubiquinone is also shown for the plant complex I. **b** The plant specific P1 protein is compared with NUNM from the *Y. lipolytiva* complex I (NDUFB5 with human nomenclature). NDUFB5 was previously shown to be absent in plants[23], the two proteins therefore share no sequence homology at all. Still, their overall structure is similar with the exception of the transverse N-ter helix which is absent. P1/NDUFB5 are shown in red and B14.5b, shown as a benchmark, is in blue.

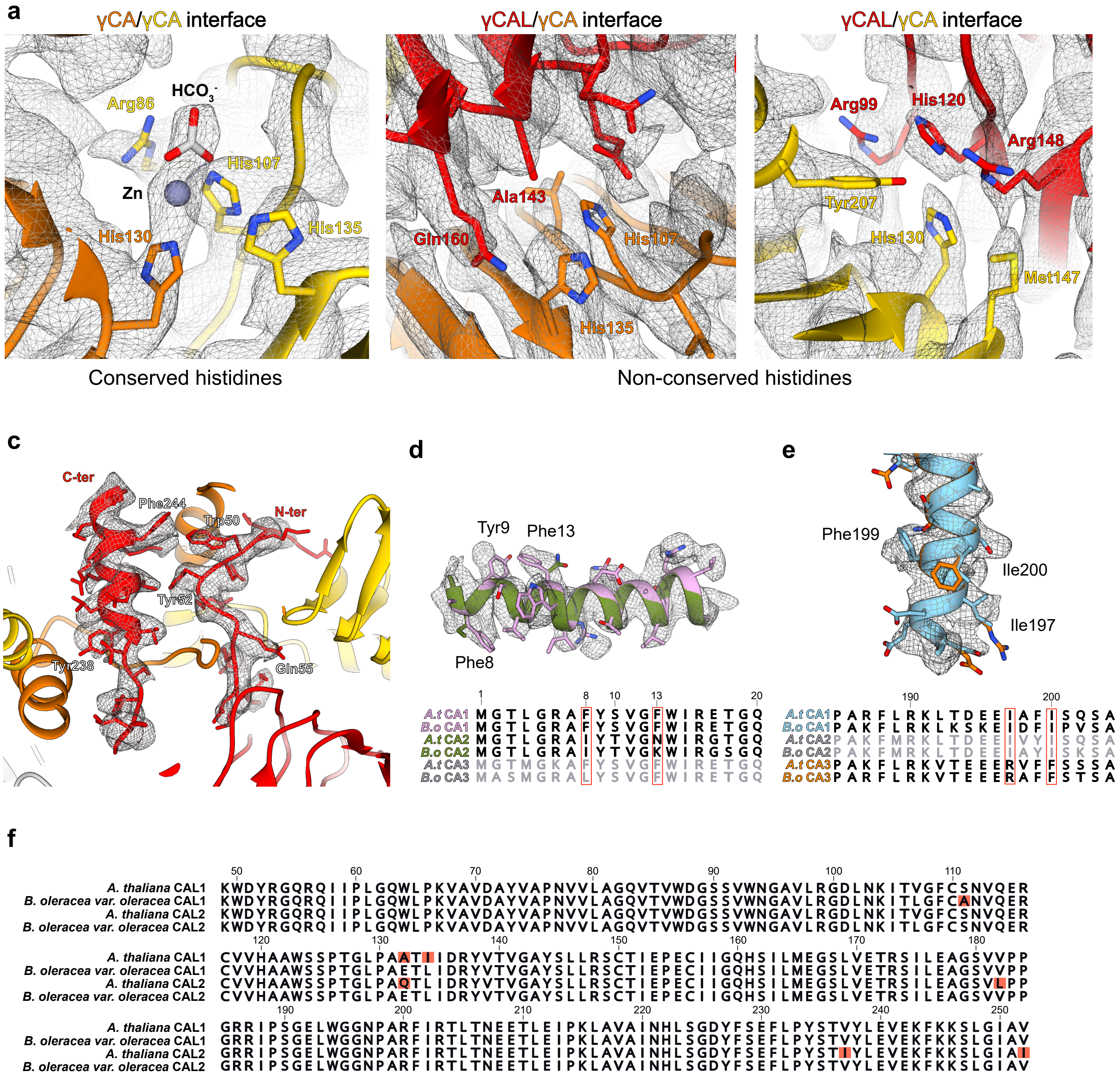

Supplementary Figure 6. Identification of carbonic anhydrase subunits

a Densities of three subunit interfaces are shown, highlighting the presence of the zinc and the HCO_3^- only at the interface of the two γCA subunits. The mutate histidines, to Ala143 and Arg148, in the γCAL are also shown. The distinction between γCA and γCAL was done based on N and C-ter tails as well as conserved histidine residues, identification of the γCAL is shown in **b**. Models in their respective densities and sequence alignment are presented for the part that contributed to identify each of the subunit. Both possibilities are displayed and alignments, of both *A. thaliana* sequences and *B. oleracea var oleracea* (wild-cabbage), are color-coded in accordance to the models. **c** For the γCA subunits, for chain p, residues Phe8 and Phe13 allowed to determine that it was γCA1 or 3 and not γCA2. **d** Residue Ile200 and Ile197, which would be a Phe and an Arg in γCA3, were used to distinguish between γCA1 and γCA3, confirming that the subunit is indeed γCA1. Still for the second γCA (chain q) the scant density did not allow to clearly distinguish between γCA1 and γCA2, and was therefore built as γCA1. **e** Alignment of the complete γCAL1 and γCAL2 sequences (excluding target sequence) of *A. thaliana* sequences and *B. oleracea var oleracea* (wild-cabbage) are displayed to illustrate the sequence homology between Arabidopsis and other plants of the Brassica family, here 98% pairwise identity and 99.8% BLSM62 pairwise positive. Given the high similarity of the two proteins (only 7 different aa highlighted in red, excluding the mitochondrial target sequence) and previous genetic screens[20] both γCAL1 and γCAL2 could be there and γCAL1 was built in the density.

Name	Uniprot ID	Chain ID	Size	Modeled Residues	Comments/ligand	AGI	*H. sapiens*	*E.coli*
**51 kDa**	Q9FNN5	A	486	53-481	FMN + FeS	At5g08530	NDUFV1	NuoF
**24 kDa**	O22769	B	255	31-249	FeS	At4g02580	NDUFV2	NuoE
**75 kDa**	Q9FGI6	C	748	50-742	3*FeS	At5g37510	NDUFS1	NuoG
**TYKY/23 kDa**	Q9FX83	D	222	45-221	2*FeS	At1g16700, At1g79010	NDUFS8	NuoI
**PSST/20 kDa**	Q42577	E	218	65-217	FeS	At5g11770	NDUFS7	NuoB
**Nad9m/30 kDa**	Q95748 *m*	F	190	8-185		AtMg00070	NDUFS3	NuoC
**Nad7m/49 kDa**	P93306 *m*	G	394	11-392		AtMg00510	NDUFS2	NuoD
**Nad1m**	P92558 *m*	H	325	7-324		AtMg00516/AtMg01120/	ND1m	NuoH
**Nad2m**	O05000 *m*	I	499	17-497		AtMg00285/AtMg01320	ND2m	NuoN
**Nad3m**	P92533 *m*	J	119	2-34/55-117		AtMg00990	ND3m	NuoA
**Nad4Lm**	Q04614 *m*	K	100	2-86		AtMg00650	ND4Lm	NuoK
**Nad4m**	P93313 *m*	L	495	12-493		AtMg00580	ND4m	NuoM
**Nad5m**	P29388 *m*	M	669	4-638		AtMg00513/AtMg0060/A	ND5m	NuoL
**Nad6m**	P60497 *m*	N	205	2-75/121-195		AtMg00270	ND6m	NuoJ
**18 kDa**	Q9FJW4	O	154	39-149		At5g67590	NDUFS4	
**13 kDa**	Q9M9M6	P	110	38-108		At3g03070	NDUFS6	
**B8**	Q9FIJ2	Q	97	3-94		At5g47890	NDUFA2	
**B13**	Q9FLX7	R	169	14-142		At5g52840	NDUFA5	
**B14.5a**	Q9SD78	S	131	29-129		At5g08060	NDUFA7	
**39 kDa**	Q9SK66	T	402	55-382	NADPH	At2g20360	NDUFA9	
**DAP13/B17.2**	Q9M9M9	U	159	32-153		At3g03100	NDUFA12	
**MWFE**	Q9C9Z5	V	65	2-58		At3g08610	NDUFA1	
**B9**	Q9ZPY5	W	65	4-40		At2g46540	NDUFA3	
**B14**	Q9LHI0	X	133	12-103/122-132		At3g12260	NDUFA6	
**PGIV**	Q8LGE7	Y	106	8-105		At5g18800, At3g06310	NDUFA8	
**B16.6/GRIM-19**	Q8RWA7	Z	143	2-141		At1g04630, At2g33220	NDUFA13	
**NDU8/B15**	Q9SIQ8	a	71	38-60		At2g31490	NDUFB4	
**B18**	Q9SKC9	b	103	13-75		At2g02050	NDUFB7	
**20.9 kDa/MNLL**	Q84W12	c	106	2-100	Orthologue present in yeast but not in animals	At4g16450		
**B22**	Q945M1	d	117	12-77	Placed by homology in filtered density, next to ACPM2	At4g34700	NDUFB9	
**ACPM2**	O80800	e	126	42-125	Placed by homology in filtered density, next to B22	At1g65290		
**PDSW**	Q9M9B4	f	107	10-85		At1g49140, At3g18410	NDUFB10	
**NDU12/ESSS**	Q8L3S7	g	114	58-104		At3g57785, At2g42310	NDUFB11	
**AGGG**	Q8LDK3	h	69	20-47	Placed by homology in filtered density	At1g76200	NDUFB2	
**NDU9/B14.5b**	Q94AL6	i	81	7-72		At4g20150	NDUFC2	
**15 kDa/Nuos5**	Q9LZI6	j	83	4-71		At3g62790, At2g47690	NDUFS5	
**ACPM1**	P53665	k	122	45-122	Placed by homology in filtered density, next to B14	At2g44620	NDUFAB1	
**B12-1**	Q9M9R9	l	73	20-50	Placed by homology in filtered density	At1g14450	NDUFB3	
**P1/11kDa**	Q9FYF8	m	98	4-72	Functionally replaces SGDH	At1g67350		
**P2/16kDa**	Q9ZUX4	n	113	81-102	Contacts CA	At2g27730		
**CAL1**	Q9FMV1	o	252	47-248		At5g63510		
**CA1**	Q9FWR5	p	275	6-229		At1g19580		
**CA1**	Q9FWR5	q	275	6-233	Modeled as CA1 but could be a mixture of CA1 and C2	At1g19580		
**UNK1**		r	-	23	Plant specific			
**UNK2**		s	-	19	Correspond to subunit NUUM in *Y.lipolytica*			
**GLDH**	Q9SU56	z	610	113-605	Only present in the assembly intermediate, placed in filtered density	At3g47930		

<u>Supplementary Table 1. List of proteins identified</u>

Complete list of the 45 proteins constituting the plant mitochondrial complex I. The proteins are colored by conservation with the bacterial complex I (blue) other mitochondrial complex I (yellow) or specific to the plant complex I (red). B14.7 and ASHI proteins, plant orthologues of NDUFA11 and NDUFB8 respectively were not observed but found in the mass-spectrometry data suggesting their presence in plant complex I. *m* designate proteins encoded by the mitochondrial genome. Full list of proteins identified is provided in Supplementary Data 1.

Cryo-EM data collection, refinement and validation statistics

	#1 Full Complex I (EMD-11614) (PDB 7A23)	#2 Focused Complex I (EMD-11513)	#3 Assembly intermediate with GLDH (EMD-11615) (PDB 7A24)
**Data collection and processing**			
Magnification		36,000	
Voltage (kV)		200	
Electron exposure (e–/Å^2)		45	
Defocus range (μm)		-0.5 to -2.5	
Pixel size (Å)		1.11	
Symmetry imposed		C1	
Initial particle images (no.)		650,844	
Final particle images (no.)	65,018		36,513
Map resolution (Å)	3.7	3.4	3.8
FSC threshold	0.143	0.143	0.143
Map resolution range (Å)	2.8 to 8	2.8 to 8	2.8 to 8
**Refinement**			
Initial model used (PDB code)		6RFR, 6G2J	
Model resolution (Å)			
FSC threshold			
Model resolution range (Å)	2.8 to 8	2.8 to 8	2.8 to 8
Map sharpening *B* factor (Å^2)	-88	-139	-78
Model composition			
Non-hydrogen atoms	57,976		49,510
Protein residues	7,354		6,253
Ligands	BCT:1		BCT:1
	ZN:2		ZN:2
	CDL:3		CDL:1
	T7X:2		T7X:2
	PEV:1		PEV:1
	U10:1		U10:1
	NDP:1		NDP:1
	FES:2		FES:2
	SF4:6		SF4:6
	FMN:1		FMN:1
B factors (Å^2)			
Protein (min./max./mean)	11.24/235.32/57.70		0.35/836.53/68.08
Ligand (min./max./mean)	16.31/49.46/23.58		16.31/49.46/26.71
R.m.s. deviations			
Bond lengths (Å)	0.008		0.010
Bond angles (°)	1.069		1.163
Validation			
MolProbity score	2.46		2.7
Clashscore	23.96		22.55
Poor rotamers (%)	1.07		2.62
Ramachandran plot			
Favored (%)	89.30%		90.09%
Allowed (%)	10.52%		9.57%
Disallowed (%)	0.18%		0.34%

Supplementary Table 2.
