# Supplementary material for: Specific features and assembly of the plant mitochondrial complex I revealed by cryo-EM
Source: Nat Commun. 2020 Oct 15;11:5195. doi: 10.1038/s41467-020-18814-w (PMC7567890; doi:10.1038/s41467-020-18814-w)
Supplement: Supplementary file 3 — Descriptions of Additional Supplementary Files [file 41467_2020_18814_MOESM3_ESM.pdf]

Descriptions of Additional Supplementary Files

Supplementary Data 1

Description: Mass-spectrometry data. Proteins identified by mass spectrometry are presented. Proteins that were built into the final model are highlighted in blue. Data were searched against the TAIR A. thaliana database and are shown here. Data were that searched against the home-made Brassica (B. oleracea var oleracea) and database extracted from UniProtKB (Swissprot+TrEMBL) are displayed in the "Brassica" sheet.
