# Supplementary material for: Specific features and assembly of the plant mitochondrial complex I revealed by cryo-EM
Source: Nat Commun. 2020 Oct 15;11:5195. doi: 10.1038/s41467-020-18814-w (PMC7567890; doi:10.1038/s41467-020-18814-w)
Supplement: Supplementary file 5 — Reporting Summary [file 41467_2020_18814_MOESM5_ESM.pdf]

nature research

Corresponding author(s): Yaser HASHEM & Florent WALTZ

Last updated by author(s): Sep 3, 2020

Reporting Summary

Nature Research wishes to improve the reproducibility of the work that we publish. This form provides structure for consistency and transparency in reporting. For further information on Nature Research policies, see Authors & Referees and the Editorial Policy Checklist .

Statistics

For all statistical analyses, confirm that the following items are present in the figure legend, table legend, main text, or Methods section.

n/a	Confirmed	
x		The exact sample size (*n*) for each experimental group/condition, given as a discrete number and unit of measurement
	x	A statement on whether measurements were taken from distinct samples or whether the same sample was measured repeatedly
x		The statistical test(s) used AND whether they are one- or two-sided<br>*Only common tests should be described solely by name; describe more complex techniques in the Methods section.*
x		A description of all covariates tested
x		A description of any assumptions or corrections, such as tests of normality and adjustment for multiple comparisons
x		A full description of the statistical parameters including central tendency (e.g. means) or other basic estimates (e.g. regression coefficient) AND variation (e.g. standard deviation) or associated estimates of uncertainty (e.g. confidence intervals)
x		For null hypothesis testing, the test statistic (e.g. *F*, *t*, *r*) with confidence intervals, effect sizes, degrees of freedom and *P* value noted<br>*Give P values as exact values whenever suitable.*
x		For Bayesian analysis, information on the choice of priors and Markov chain Monte Carlo settings
		For hierarchical and complex designs, identification of the appropriate level for tests and full reporting of outcomes
x		Estimates of effect sizes (e.g. Cohen's *d*, Pearson's *r*), indicating how they were calculated

Our web collection on statistics for biologists contains articles on many of the points above.

Software and code

Policy information about availability of computer code

Data collection	Data collection was performed on a Talos Artica instrument (Thermofisher) at 200 kV using the EPU for Life Science v2.4 software (Thermofisher) for automated data acquisition. Data were collected at a nominal underfocus of -0.5 to -2.5 µm at a magnification of 36,000 X yielding a pixel size of 1.11 Å
Data analysis	MotionCorr2, Gctf v1.06, RELION 3.0, ResMap 1.95, Swiss-model, Coot 0.9, Phenix 1.17, Chimera 1.13.1, ChimeraX 0.9

For manuscripts utilizing custom algorithms or software that are central to the research but not yet described in published literature, software must be made available to editors/reviewers. We strongly encourage code deposition in a community repository (e.g. GitHub). See the Nature Research guidelines for submitting code & software for further information.

Data

Policy information about availability of data

All manuscripts must include a data availability statement. This statement should provide the following information, where applicable:

- Accession codes, unique identifiers, or web links for publicly available datasets
- A list of figures that have associated raw data
- A description of any restrictions on data availability

The cryo-EM maps of plant mitochondrial complex I have been deposited at the Electron Microscopy Data Bank (EMDB): EMD-11614 for the mature complex, EMD-11513 for the focused reconstruction, EMD-11615 for the assembly intermediate. The corresponding atomic models been deposited in the Protein Data Bank (PDB) under the accession 7A23 for the mature complex and 7A24 for the assembly intermediate. Mass spectrometric data have been deposited to the ProteomeXchange Consortium via the PRIDE partner repository with the dataset identifier PXD017847 and 10.6019/PXD017847.

Field-specific reporting

Please select the one below that is the best fit for your research. If you are not sure, read the appropriate sections before making your selection.

☒ Life sciences ☐ Behavioural & social sciences ☐ Ecological, evolutionary & environmental sciences

For a reference copy of the document with all sections, see nature.com/documents/nr-reporting-summary-flat.pdf

Life sciences study design

All studies must disclose on these points even when the disclosure is negative.

Sample size	One biological sample was analyzed, which resulted in the mature and assembly intermediate of complex I
Data exclusions	No data were excluded
Replication	Purification experiments were repeated in the same conditions and always yielded the same complexes when analyzed by cryo-EM and mass spectrometry.
Randomization	Not relevant to our study. Cauliflower for mitochondria and complex I purification were randomly chosen, but no randomization is applied during cryo-em data collection, processing, and interpretation.
Blinding	Not relevant to our study since it requires prior knowledge of the complexe of interest.

Reporting for specific materials, systems and methods

We require information from authors about some types of materials, experimental systems and methods used in many studies. Here, indicate whether each material, system or method listed is relevant to your study. If you are not sure if a list item applies to your research, read the appropriate section before selecting a response.

Materials & experimental systems

n/a	Involved in the study
☒	☐ Antibodies
☒	☐ Eukaryotic cell lines
☒	☐ Palaeontology
☒	☐ Animals and other organisms
☒	☐ Human research participants
☒	☐ Clinical data

Methods

n/a	Involved in the study
☒	☐ ChIP-seq
☒	☐ Flow cytometry
☒	☐ MRI-based neuroimaging
